# Correlations between schizophrenia and lichen planus: a two-sample bidirectional Mendelian randomization study

**DOI:** 10.3389/fpsyt.2023.1243044

**Published:** 2023-09-13

**Authors:** Guan-Yu Chen, Ling-ling Fu, Bin Ye, Man Ao, Ming Yan, Hong-Chao Feng

**Affiliations:** ^1^College of Stomatology, Guizhou Medical University, Guiyang, China; ^2^Department of Oral and Maxillofacial Surgery, Guiyang Hospital of Stomatology, Guiyang, China; ^3^Department of Oral and Maxillofacial Surgery, University Medical Center Hamburg-Eppendorf, Hamburg, Germany

**Keywords:** Mendelian randomization study, schizophrenia, lichen planus, causal relationship, mental illness

## Abstract

**Background:**

Several existing studies have shown a correlation between schizophrenia and lichen planus (LP). However, the causality of this relationship remains uncertain. Thus, this study aimed to examine the causal association between schizophrenia and LP.

**Methods:**

A two-sample Mendelian randomization (MR) study was carried out to investigate whether schizophrenia is causally related to LP and vice versa, and genetic variants in this study were taken from previous genome-wide association studies. We used the inverse variance weighted (IVW) method as the main analysis. Furthermore, several sensitivity analyses were performed to assess heterogeneity, horizontal pleiotropy, and stability.

**Results:**

Our results show that schizophrenia has a protective effect on LP (OR = 0.881, 95%CI = 0.795–0.975, *p* = 0.015). Conversely, we observed no significant relationship between LP and schizophrenia in reverse MR analysis (OR = 0.934, 95%CI = 0.851–1.026, *p* = 0.156).

**Conclusion:**

Our two-sample Mendelian randomization study supports a significant causal relationship between LP and schizophrenia and finds that schizophrenia can reduce the incidence of LP. This is in contrast to previous findings and provides new insights into the relationship between LP and schizophrenia, but the exact mechanism needs further investigation.

## 1. Introduction

Schizophrenia, a chronic mental disorder with a multifactorial neurodevelopmental etiology (1) affects almost one in 100 individuals worldwide (2). Research indicates that patients with schizophrenia may experience fragmented thinking, emotions, behaviors, and perceptions, along with discordance between their mental processes and the surrounding environment. The disease typically follows a protracted course and is prone to relapses, leading to significant impacts on patients’ physical and mental well-being, as well as their social functioning. Consequently, long-term treatment is necessary (3–5). Currently, the specific causes of schizophrenia remain unclear. However, it is widely believed that genetics, environmental factors, and dopamine receptors play pivotal roles in its development (6). In addition, schizophrenia is often accompanied by comorbidities such as depression, anxiety, sleep, circadian rhythm disorders (7), and even suicidal tendencies in patients. Patients with schizophrenia comorbidity may suffer from physical health problems, including skin disorders (8), due to decreased self-care and social isolation. Therefore, schizophrenia has been recognized as a lifelong condition associated with social challenges, constituting a significant global concern (9).

Lichen planus (LP) is an immune-inflammatory disease with an unknown etiology that affects the skin (10), mucous membranes, and skin appendages. It typically manifests in middle-aged individuals and presents as a characteristic rash of purplish-red, polygonal, flat papules, and plaques (11). LP is a chronic and recurring condition that currently lacks sufficient diagnostic tools and effective treatment protocols. Lesions of LP in the oral mucosa can hinder the patient’s ability to eat, while those on exposed skin can significantly disrupt their social and daily activities. Oral lichen planus, a potentially malignant disorder, has no known cure and carries the risk of progressing into oral squamous cell carcinoma (12, 13). Thus, early detection and appropriate management of oral lichen planus are crucial in preventing the development of oral cancer. However, there is a lack of clinical screening techniques for LP that are both non-invasive and provide early and objective detection.

Mental stressors have the potential to induce atypical alterations in inflammatory factors, leading to immune dysfunction, which may contribute to the development of LP, as immune-mediated mechanisms of LP are well recognized (14). Some studies have also suggested that people with schizophrenia may have a higher risk of autoimmune diseases (15). In addition, schizophrenia and LP may share some inflammatory pathways. For example, some studies have found that inflammatory mediators such as tumor necrosis factor-alpha (TNF-α) and interleukin-6 (IL-6) may play an important role in both disorders (16, 17). LP may be accompanied by psychiatric comorbidities, and multivariable analysis has demonstrated a positive correlation between LP and an increased likelihood of depression and anxiety (18). Furthermore, schizophrenia has been found to significantly influence the development and severity of LP (19), with research indicating a correlation between schizophrenia and the occurrence of oral lichen planus (20–22). The pathogenesis of LP is influenced by various factors, including individual patients and their potential etiology. It has been established that anxiety, stress, and depression are associated with LP (23). Studies have shown that patients with LP are more susceptible to depression compared to those in the control group, highlighting the significant burden of LP on patients and its contribution to psychological distress and mental illness (24, 25).

**Figure 1:**
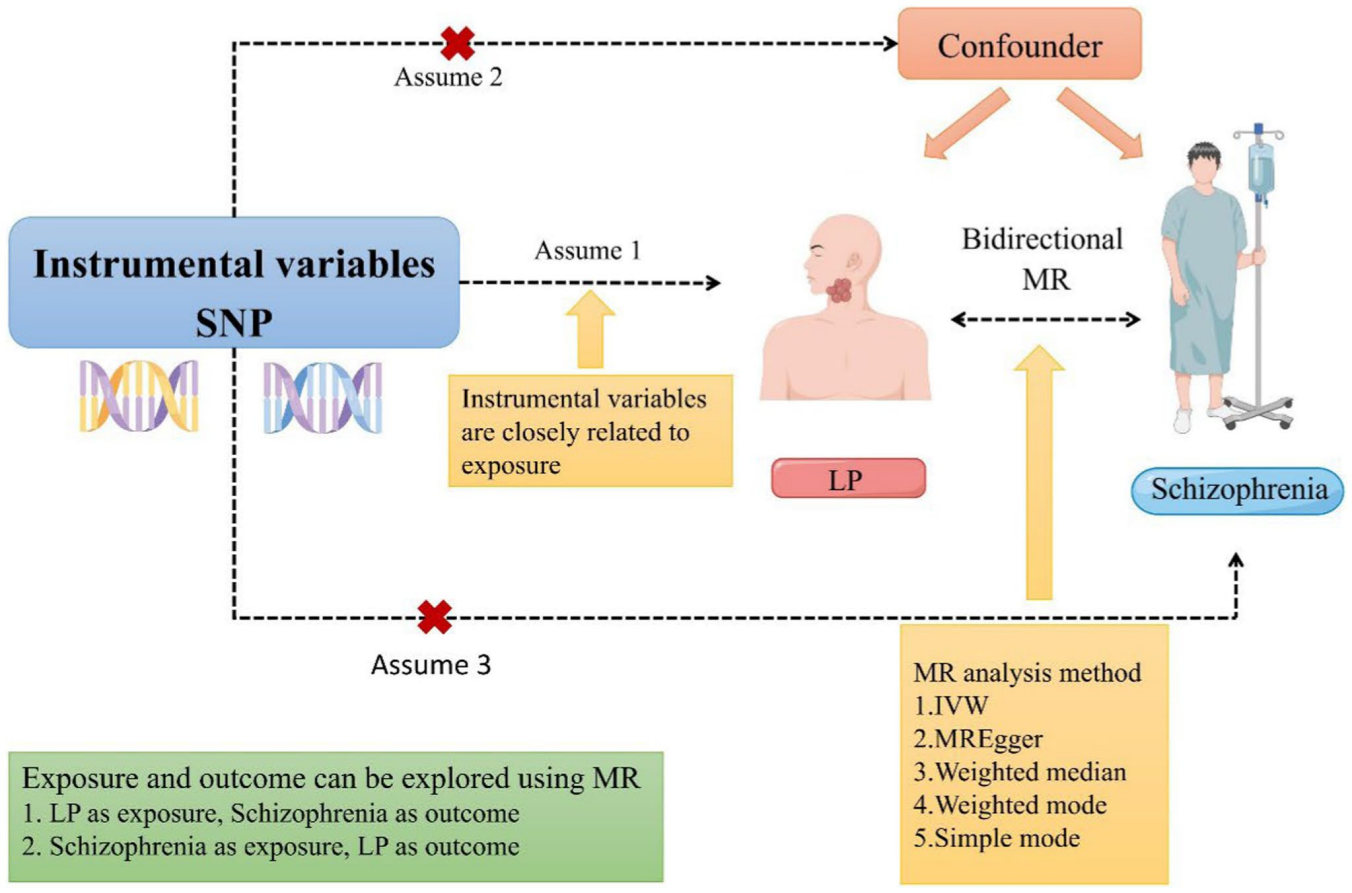
A schematic representation of a two-sample Mendelian randomization study investigating the relationship between LP and schizophrenia has been proposed. This design postulates a bidirectional association between LP and schizophrenia while disregarding any association with confounding variables. It is plausible for genetic variations to influence schizophrenia through LP and vice versa (MR, Mendelian randomization; SNP, single nucleotide polymorphism; LP, Lichen planus; and IVW, inverse variance-weighted).

To investigate the bidirectional causal relationship between schizophrenia and LP in the present study, we conducted a two-sample bidirectional Mendelian randomization study. To evaluate the causality of potential exposure pathways, the use of genome-wide association data (GWAS) may be considered as a way to overcome the limitations of observational studies. An instrumental variable used in MR is genetic variation. The purpose is to develop a causal link between exposure and outcome using genetic variation as an instrument (26). When randomized controlled trials are not feasible, MR serves as an alternative approach for investigating potential causal links between exposure and outcome. Therefore, we employed a bidirectional MR design to examine the bidirectional causal association between schizophrenia and LP (Figure 1), thereby addressing an existing research gap and investigating the effects of LP on individuals with schizophrenia.

## 2. Materials and methods

### 2.1. Data sources

From a previous meta-analysis of genetic data conducted by Trubetskoy et al. (27) that included Europeans, East Asians, African Americans, and Latinos to conform a sample that contained 76,755 schizophrenia cases and 243,649 controls, we screened the data to select 53,386 cases and 77,258 controls of European ancestry for the present study. The LP dataset (GWAS ID finn-b-L12_LICHENPLANUS) utilized in this study was sourced from the IEU Open GWAS database, which comprises European samples consisting of 1,865 cases and 212,242 controls. The Open GWAS database is an open-source platform that adheres to rigorous quality control measures. Furthermore, the LP dataset from the IEU Open GWAS database was included in the European samples, which encompassed 1,865 cases and 212,242 controls. The Open GWAS database is a publicly accessible and open-source database that adheres to rigorous quality control measures.

### 2.2. Instrumental variables

The assumptions underlying MR are highly stringent and encompass three key tenets: the relevance assumption posits that the strength of instrumental variables can be measured through the use of the F statistic, with a value greater than 10 indicating strong instruments. The independence assumption holds that other variables are not significantly associated with the outcome, while the exclusive assumption asserts that the instrumental variables are independent of other confounding factors (28). We extracted IV according to the standard of *p* < 5 × 10^−8^ in reverse MR, and *p* < 5 × 10^−6^ in reverse MR. To avoid linkage disequilibrium (LD) bias, LD with significant SNPs associated with exposure factors must fulfill the following conditions: *r*^2^ < 0.001 and a genetic distance of 10,000 kb. In the event of an *F* statistic exceeding 10, the presence of weak instrumental variables bias can be discounted (29, 30). In this study, the *F* statistic and the variance explained by instrumental variables (IVs) were calculated with the formulas proportionated in the Supplementary material.

### 2.3. Statistical analysis

The study employed a two-sided analysis utilizing R software (version 4.2.1), as well as two-sided analyses utilizing MRPRESSO (1.0) and Two Sample MR (0.5.6) packages (31, 32). The inverse variance-weighted (IVW) method was utilized as the primary analytical technique (33). While the IVW method is commonly used, it assumes that all instrumental variables are dependable, and if one SNP fails to meet the assumptions of instrumental variables, it may lead to a biased outcome (34). Furthermore, assuming all genetic variants are valid, the IVW method yields MR estimates of greater validity, albeit with increased susceptibility to pleiotropy bias. To augment the comprehensiveness and robustness of our analysis, we employed the MR-Egger method (33), weighted mode method (35), simple mode method, and weighted median method in conjunction with the IVW method. Additionally, we conducted sensitivity analyses using the MR-PRESSO method to estimate horizontal pleiotropy and instrument strength (36, 37).

## 3. Results

### 3.1. Causal effects of schizophrenia on LP

**Figure 2:**
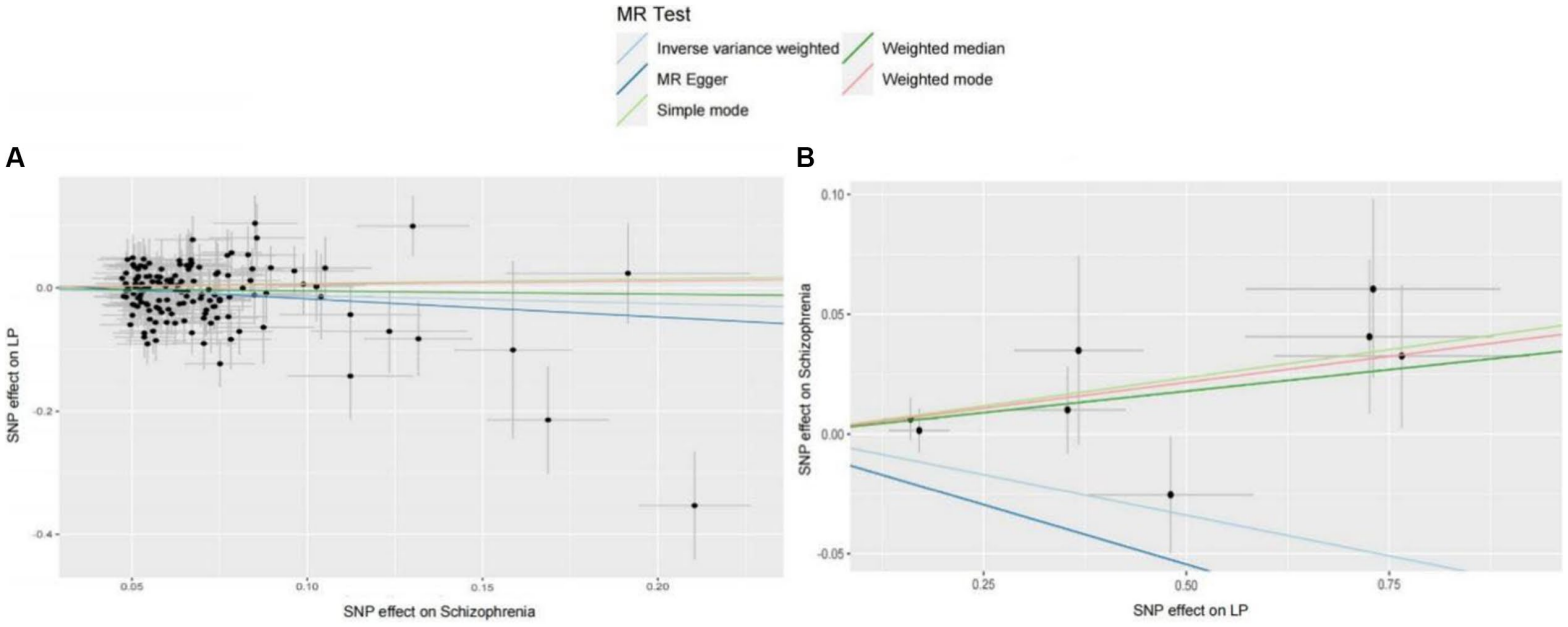
**(A)** MR estimates the causal relationship between schizophrenia and LP. The log odds ratio of risk is shown, and five different methods (IVW, MR-Egger, weighted median, weighted mode, and simple mode) were used. The scattered plot of SNPs associated with schizophrenia and their risk on LP. **(B)** MR estimates the causal relationship between LP and schizophrenia. The log odds ratio of risk is shown, and five different methods (IVW, MR-Egger, weighted median, weighted mode, and simple mode) were used. The scattered plot of SNPs associated with LP and their risk on schizophrenia.

**Figure 3:**
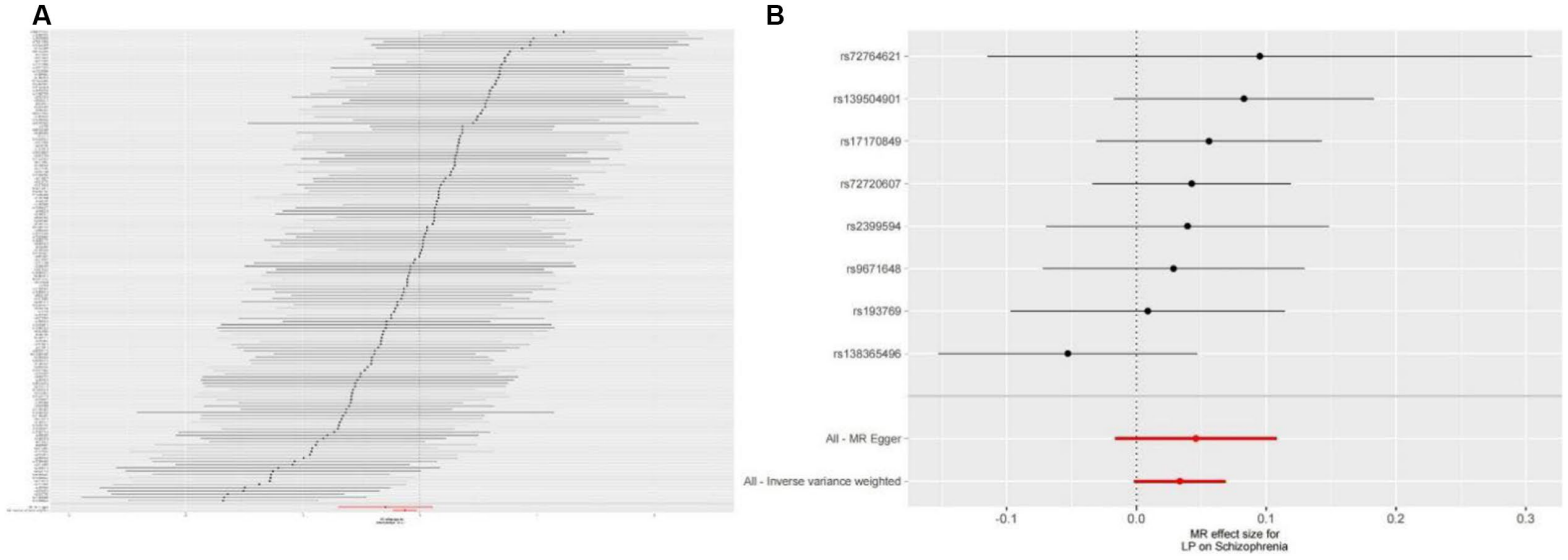
**(A)** Forest plots of causal effects of schizophrenia-associated SNPs on LP. **(B)** Forest plots of causal effects of LP-associated SNPs on schizophrenia.

**Figure 4:**
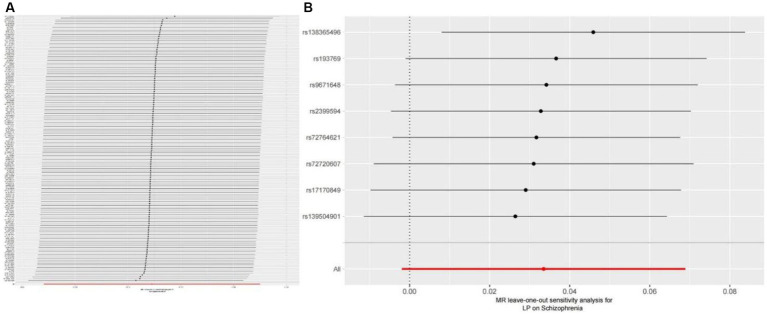
**(A)** Leave-one-out plot for the genetic causal association between schizophrenia and LP. **(B)** Leave-one-out plot for the genetic causal association between schizophrenia and LP.

We extracted 154 SNPs using schizophrenia as the exposure and LP as the outcome (Supplementary Tables S1, S2). The results showed IVW (OR = 0.881, 95%CI = 0.795–0.975, *p* = 0.015), MR-Egger (OR = 0.746, 95%CI = 0.497–1.118, *p* = 0.158), and weighted median (OR = 0.950, 95%CI = 0.829–1.089, *p* = 0.462; Table 1). This two-sample MR study found that schizophrenia was causally associated with LP (Figure 2A). Moreover, MR-Egger regression results show that there is no horizontal pleiotropy in our analysis (intercept = 0.011, *p* = 0.407; Table 2; Figure 3A). Each black dot represents an SNP, the *x*-axis represents the effect of increased SNP on exposure, the *Y*-axis represents the effect of increased SNP on the outcome, and the slope of each line represents the potential causal correlation Each black dot represents an SNP of each method. In addition, according to the leave-one-out analysis, there was no significant difference in the causal association between schizophrenia and LP, which indicated that no single SNP affected the causal estimation results (Figure 4A). Funnel plots for the visualization are shown in Supplementary Figure 1.

**Table 1:** An estimate of the relationship between genetically instrumented LP and schizophrenia based on MR estimates.

Exposure	Outcome	Beta	SE	SNPs	Method	OR	95%CI	*p* value
Schizophrenia	LP	−0.127	0.052	145	IVW	0.881	0.795–0.975	0.015
		−0.294	0.207	145	MR-Egger	0.746	0.497–1.118	0.158
		−0.051	0.070	145	Weighted median	0.950	0.829–1.089	0.462
		0.055	0.207	145	Weighted mode	1.057	0.704–1.586	0.791
		0.071	0.215	145	Simple mode	1.074	0.704–1.638	0.741
LP	Schizophrenia	−0.068	0.048	11	IVW	0.934	0.851–1.026	0.156
		−0.100	0.094	11	MR-Egger	0.905	0.752–1.089	0.318
		0.036	0.024	11	Weighted median	1.036	0.990–1.085	0.128
		0.047	0.026	11	Weighted mode	1.044	0.993–1.098	0.122
		0.043	0.030	11	Simple mode	1.048	0.988–1.111	0.122

MR, Mendelian randomization; SE, Standard error; IVW, Inverse variance-weighted; OR, Odds ratio; CI, Confidence interval; and LP, Lichen planus.

**Table 2:** Heterogeneity and MR-Egger test for directional pleiotropy.

Exposure	Outcome	Heterogeneity			MR-Egger test		
		Q	df	*p* value	Intercept	SE	*p* value
Schizophrenia	LP	166.114	143	0.100	0.011	0.013	0.407
LP	Schizophrenia	4.675	7	0.700	−0.005	0.010	0.655

MR, Mendelian randomization; LP, Lichen planus; Q, Heterogeneity statistic Q; df, Degree of freedom; and SE, Standard error.

### 3.2. Causal effects of LP on schizophrenia

We also carried out reverse-MR analysis to avoid the potential effects of reverse causality. LP was taken as exposure, and schizophrenia was taken as outcome, eight SNPs were extracted (Supplementary Tables S1, S2), showing IVW (OR = 0.934, 95%CI = 0.851–1.026, *p* = 0.156), weighted median (OR = 0.985, 95%CI = 0.990–1.085, *p* = 0.128), and according to MR-egger (OR = 1.036, 95%CI = 0.752–1.089, *p* = 0.318), there is no significant association between LP and schizophrenia (Table 1; Figures 2B, 3B). In the MR-Egger analysis, there was no horizontal pleiotropy of LP on schizophrenia risk: intercept = −0.005, *p* = 0.65 (Table 2). As a result of the leave-one-out analysis, several obvious abnormalities were not observed (Figure 4B). Therefore, the MR findings do not support a causal role for LP in schizophrenia.

## 4. Discussion

In this study, bidirectional MR was used to analyze published GWAS datasets to determine whether schizophrenia and LP have a bidirectional causal relationship. According to our results, we found that schizophrenia can reduce the incidence of LP. However, our findings do not support a causal relationship between genetic susceptibility to LP and increased risk of schizophrenia. Moreover, as a result of the sensitivity analysis, we were able to validate that the study results were robust and reliable.

At present, there is no clear etiology or pathogenesis of LP, and there has been considerable discussion about the relationship between schizophrenia and LP, but it still bears some controversy. As soon as some patients are exposed to mental stress, the condition becomes worse regarding lesion size, form, and pain (38). However, our reverse MR analysis contradicted those of prior observational studies (5), this may be because physician-dependent assessments can vary widely in observational studies. In addition, current studies have not been able to establish a direct cause-and-effect relationship between schizophrenia and LP.

Schizophrenia is a multifaceted psychiatric disorder with a diverse etiology, encompassing a cluster of psychiatric disorders of indeterminate origin. Currently, antipsychotic medications are the primary treatment modality for schizophrenia. Certain clinical studies indicate that the combination of antipsychotic drugs with nonsteroidal anti-inflammatory drugs and anti-inflammatory agents can ameliorate symptoms in individuals with schizophrenia (39). Over time, research has revealed intricate connections between the immune system, inflammation, and alterations in mood, cognition, and behavior. Cytokines, which are closely linked to neuroinflammatory mechanisms, can impact neurotransmission involving dopamine, 5-HT, and norepinephrine, resulting in psychiatric or depressive symptoms (40). One study suggests that abnormal functioning of the hypothalamic–pituitary–adrenal (HPA) axis may be involved in the pathogenesis of schizophrenia (41), leading to the development of several negative clinical conditions (e.g., suicidal behavior), but there is also another study that suggests that HPA-axis activity is associated with negative clinical outcomes regardless of the presence of psychiatric conditions (42). Therefore, we suggest that schizophrenia is a complex disorder whose pathogenesis involves the interaction of several factors, of which the HPA axis may be one of the important ones. In addition, elevated inflammatory mediators in the periphery and brain may be related to the pathogenesis of schizophrenia, as increased inflammatory mediators may lead to neurotransmitter disorders, abnormal neuronal function, and neurological damage, which in turn may affect the normal functioning of the brain (43). The study by Serafini et al. (44) demonstrated that higher average concentrations of inflammatory mediators were found in both the periphery and the brain of individuals at risk of suicide compared to non-suicidal subjects. A multitude of investigations have concentrated on the correlation between cytokines and schizophrenia, aiming to pinpoint potential etiologies and establish efficacious interventions to alleviate the disease’s impact. An increasing number of studies have attempted to prospectively identify populations at ultra-high risk for schizophrenia to enhance their protective factors and provide effective early intervention methods to delay or reduce the onset of schizophrenia and improve its prognosis (45). Because of the lack of biomarkers for ultra-high-risk of schizophrenia, the most effective way to recognize schizophrenia is through prodromal symptoms and recognition tools that take into account the impact of multiple risk factors including family history, family and psychosocial stressors. Due to abnormal mental activity, low volitional activity, long duration of illness, laziness, and reduced salivary secretion caused by some antipsychotic drugs, schizophrenic patients are prone to accumulation of plaque, calculus, and soft scale in their oral cavity, which eventually leads to an increase in the incidence of oral diseases. Oral problems not only greatly affect patients’ eating and speech functions, but also interfere with social and psychological activities. While our MR study findings do not provide evidence for a causal relationship between LP and schizophrenia, it is incontrovertible that individuals with LP experience significant psychological distress during their illness (46). Additionally, prior research indicates that emotional stress, including insomnia and anxiety, is prevalent among LP patients and may contribute to the onset and progression of the condition (47). Consequently, we contend that addressing the mental stressors underlying LP is an essential component of the treatment regimen for individuals with schizophrenia, in conjunction with pharmacological interventions.

Lichen planus is an immune-inflammatory skin disease, and inflammatory skin disease will appear as a rash on the skin mucosa, and oral lichen planus is an immune-mediated condition affecting the mucosa of the mouth (48), often presenting with white striae, and occasionally accompanied by ulcers and erosions, resulting in discomfort and impaired oral intake (49). There is still no clear understanding of how LP develops but is thought to be influenced by a complex interplay of cellular immunity, genetic predisposition, environmental factors, and psychological stressors, including drug exposure, viral infections, consumption of irritating foods, and emotional distress. Current treatments for LP are mainly symptomatic, with glucocorticoids being the first-line drug of choice for the treatment of LP, and specialized treatments are lacking (50). It has been found that LP and mood disorders may be caused by a common immune activation state in which T-cell subsets change and activate interferon family cytokines. Specifically, IFN-α and IFN-γ were identified as critical immune regulators of LP and mood disorders (51). In addition, the prevalence of psychological disorders, including stress, depression, anxiety, and schizophrenia, is higher in patients with LP (52), but our results of the present MR study do not support that at the genetic level, LP increases the risk of psychotic schizophrenia, and interestingly, our MR results support that schizophrenia reduces the risk of LP. In addition, psychological changes may lead to immune dysfunction and influence the development of autoimmune diseases (53). T cells, chemokines, interleukins, and tumor necrosis factors are involved in the pathological damage process of LP. By detecting the cellular immune function and cytokine levels of patients, the condition and prognosis of LP patients can be evaluated and clinical treatment can be guided. However, further studies are needed to fully understand the specific process of immune factor-mediated LP pathogenesis and pathological development (14).

In addition, we have combined other existing studies to formulate some plausible hypotheses for our current findings. The first is the medication factors, patients with schizophrenia are usually treated with a number of antipsychotic medications that have anti-inflammatory and immunomodulatory effects (54). And another study has shown that psychiatric drugs are also one of the common medications used by people with LP (55), which could be one of the risk factors for schizophrenia to reduce the development of LP. Second, there are neurotransmitter modulation factors, and studies have shown that schizophrenia is usually associated with neurotransmitter disorders, especially dopamine and acetylcholine (56, 57). Whereas the development of LP is similarly associated with neurotransmitter disorders, we hypothesized that neurotransmitter abnormalities in patients with schizophrenia might reduce the risk of developing LP (58). In summary, mental factors have a close relationship with the occurrence and development of LP, and stress, emotional disorders, and personality traits play an important role in the pathogenesis of LP. Although our study crossed to show that schizophrenia decreases the risk of LP occurrence, there is no doubt that schizophrenia can cause great mental suffering to LP patients. Therefore, we believe that in addition to the pharmacological treatment of LP patients related to psychiatric factors, the development of LP patients’ own psychological qualities and character traits should be enhanced to achieve better treatment outcomes and improve the prognosis of the disease.

The utilization of genetic variants in MR enables the estimation of health outcomes associated with phenotypes that are influenced by said variants. MR, an established genetic epidemiology technique, emulates a lifelong randomized controlled trial by leveraging natural genetic variation. The IVW method, for instance, concentrates on the integration of numerous genetic variants to ascertain the causal relationship between exposure factors and outcome outcomes. The present study’s outstands are as follows. Research on psychiatric disorders through MR has increased in recent years (59–61), also because, by randomizing groups, MR allows potential confounders to be evenly distributed between test and control groups. This can minimize the interference of other factors on the effect of interventions on mental illness, and MR can improve the reproducibility of the study through rigorous experimental design and methodology, which is highly scientific and reliable and can provide strong evidence to support the assessment of the effect of interventions on mental illness (62). This study represents a pioneering effort in examining the association between LP and schizophrenia through the utilization of MR methods, thereby broadening the scope of conventional observational studies. Furthermore, the reliability of this study is underscored by the use of multiple MR methods that yielded largely congruent results. The utilization of MR analysis confers the benefit of circumventing reverse causal associations and confounding variables, while also conserving time and resources relative to observational studies. Finally, the absence of cross-sectional pleiotropy in this study serves to affirm the soundness of our conclusions.

There are limitations to this MR study. First, our biggest limitation is that we are using a single sample of European ancestry with limited representativeness, so the extent to which our results generalize to other ancestral groups is uncertain. Second, we did not stratify the causal effects between schizophrenia and LP by gender or age. Finally, it is necessary to perform a large-scale GWAS for MR to determine more genetic variation due to the IV’s strength based on sample size. Therefore, we encourage other researchers to conduct similar studies to validate and replicate the results of our Mendelian randomization study. The generalizability of results is more convincing if multiple independent studies reach similar conclusions. In addition, the results of the Mendelian randomization study are synthesized with the results of other related studies to obtain more comprehensive and reliable conclusions.

## 5. Conclusion

In summary, our two-sample Mendelian randomization study provides strong evidence for a causal relationship between LP and schizophrenia. Surprisingly, we found that schizophrenia actually decreases the incidence of LP, which contradicts previous findings and offers new insights into this complex relationship. However, it is important to note that our analysis does not establish a causal link between genetic susceptibility to schizophrenia and an increased risk of LP. Therefore, further comprehensive investigations are needed to fully understand the limitations of our study.

## Data availability statement

The original contributions presented in the study are included in the article/Supplementary material, further inquiries can be directed to the corresponding author.

## Ethics statement

Ethical review and approval was not required for the study on human participants in accordance with the local legislation and institutional requirements. Written informed consent from the patients/participants or patients/participants’ legal guardian/next of kin was not required to participate in this study in accordance with the national legislation and the institutional requirements.

## Author contributions

G-YC: developing the conceptual framework, investigating the methodology, gathering data, and writing the original draft. L-lF: developing the conceptual framework, investigating the methodology, and gathering data. MA, BY, and MY: investigation, review, and editing. H-CF: conceptualizing, reviewing, and editing. All authors contributed to the article and approved the submitted version.

## Funding

Guizhou Provincial Health Commission provided support through its Science and Technology Fund (gzwkj2022-431). MY was supported by the Merit Scholarship of Hamburg University for International Students (No. 7238065). L-lF was supported by the Chinese Government Scholarship (CSC Scholarship; No. 202208520014).

## Conflict of interest

The authors declare that the research was conducted in the absence of any commercial or financial relationships that could be construed as a potential conflict of interest.

## Publisher’s note

All claims expressed in this article are solely those of the authors and do not necessarily represent those of their affiliated organizations, or those of the publisher, the editors and the reviewers. Any product that may be evaluated in this article, or claim that may be made by its manufacturer, is not guaranteed or endorsed by the publisher.
