# Supplementary material for: Correlations between schizophrenia and lichen planus: a two-sample bidirectional Mendelian randomization study
Source: Front Psychiatry. 2023 Sep 13;14:1243044. doi: 10.3389/fpsyt.2023.1243044 (PMC10525345; doi:10.3389/fpsyt.2023.1243044)
Supplement: Supplementary file 1 [file Data_Sheet_1.docx]

The F statistic for each single nucleotide polymorphism was computed using the formula provided.

$$F=\frac{R^{2}}{1-R^{2}}\times\frac{N-k-1}{k}（F＞10）$$

N indicates the number of GWAS-exposed samples, k indicates the number of IVs, and R^2^ represents the variance of exposure explained by each IV.

In addition, we calculated the variance of exposure explained by IVs using the following formula:

$$R^{2}=2\times(1-MAF)\times MAF\times\frac{\beta}{\mathrm{SD}}$$

$$SD=SE\times\sqrt{N}$$

MAF means the minor allele frequency, β means the effect of SNP on exposure, and SD means standard deviation, SE represents the standard error of β.
