# Supplementary material for: Correlations between schizophrenia and lichen planus: a two-sample bidirectional Mendelian randomization study
Source: Front Psychiatry. 2023 Sep 13;14:1243044. doi: 10.3389/fpsyt.2023.1243044 (PMC10525345; doi:10.3389/fpsyt.2023.1243044)
Supplement: Supplementary file 2 [file Image_1.pdf]

Supplementary Figure 1:

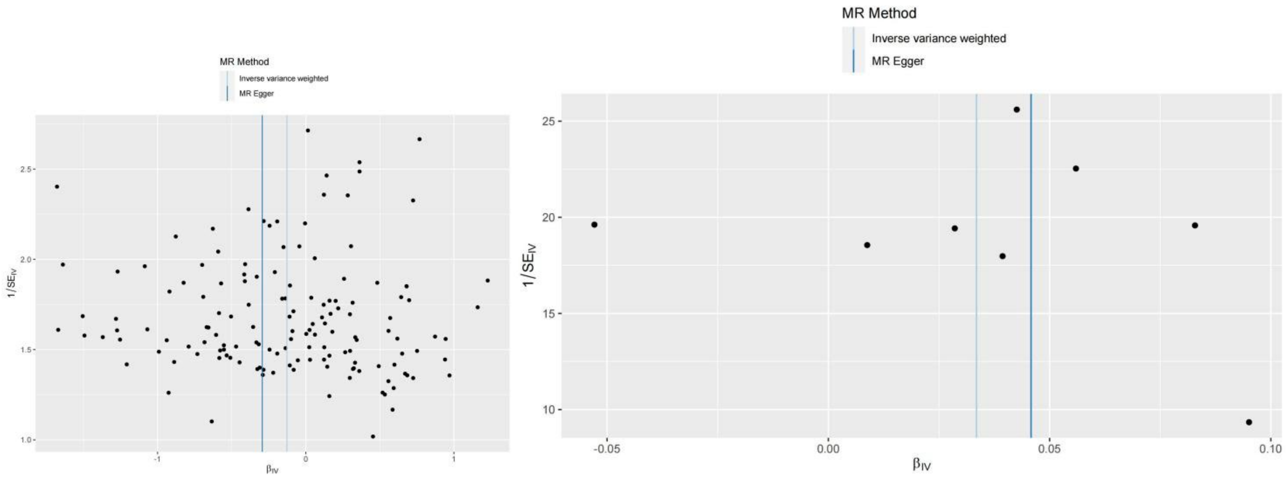

Supplementary Figure 1: Funnel plot of SNPs. On the left, is the funnel plot of the analysis between Schizophrenia and LP. On the right is the funnel plot of the analysis between LP and Schizophrenia.
