# Supplementary material for: Correlations between schizophrenia and lichen planus: a two-sample bidirectional Mendelian randomization study
Source: Front Psychiatry. 2023 Sep 13;14:1243044. doi: 10.3389/fpsyt.2023.1243044 (PMC10525345; doi:10.3389/fpsyt.2023.1243044)
Supplement: Supplementary file 3 [file Table_1.docx]

**Table S1 Instrumental variables used in MR analysis of the association between**

**schizophrenia and LP**

| SNP | chr | EA | OA | BE | eaf | se | pval |
| --- | --- | --- | --- | --- | --- | --- | --- |
| rs1000237 | 19 | A | T | 0.0732053 | 0.2661 | 0.0382 | 2.80E-16 |
| rs10035564 | 5 | G | A | 0.0668024 | 0.3212 | 0.0361 | 4.38E-13 |
| rs10086619 | 8 | G | A | 0.0722052 | 0.1203 | 0.052 | 4.97E-10 |
| rs10103330 | 8 | G | T | 0.067305 | 0.0227 | 0.1136 | 5.07E-10 |
| rs10103330 | 8 | A | T | 0.067305 | 0.2598 | 0.0388 | 5.07E-10 |
| rs10117 | 5 | A | G | -0.0549994 | 0.3498 | 0.0354 | 4.66E-10 |
| rs10876446 | 12 | C | G | 0.0540022 | 0.3398 | 0.0356 | 1.03E-08 |
| rs10957321 | 8 | A | G | 0.0475949 | 0.6279 | 0.035 | 3.48E-08 |
| rs11027839 | 11 | C | A | 0.0515038 | 0.6039 | 0.0345 | 2.40E-09 |
| rs11136325 | 8 | A | G | -0.0537967 | 0.5127 | 0.0339 | 3.05E-09 |
| rs11165867 | 1 | T | C | 0.0743034 | 0.1613 | 0.0458 | 1.30E-10 |
| rs11191580 | 10 | C | T | -0.131703 | 0.08354 | 0.0607 | 1.77E-17 |
| rs11210892 | 1 | A | G | -0.0635005 | 0.6727 | 0.0358 | 2.68E-12 |
| rs113264400 | 20 | C | T | 0.112296 | 0.1358 | 0.0493 | 2.86E-08 |
| rs11587347 | 1 | G | C | 0.103895 | 0.06487 | 0.0689 | 1.53E-12 |
| rs11664298 | 18 | A | G | 0.0773995 | 0.2168 | 0.0409 | 8.94E-13 |
| rs11693094 | 2 | T | C | -0.054403 | 0.4533 | 0.0338 | 4.29E-10 |
| rs11696755 | 20 | C | T | 0.0636962 | 0.1352 | 0.0495 | 7.26E-09 |
| rs11740474 | 5 | T | A | 0.0536962 | 0.3926 | 0.0344 | 1.13E-09 |
| rs11941714 | 4 | A | G | -0.0515957 | 0.3515 | 0.0355 | 3.07E-08 |
| rs1198588 | 1 | T | A | 0.102598 | 0.7262 | 0.0378 | 1.73E-21 |
| rs12129573 | 1 | A | C | 0.0777994 | 0.3563 | 0.0352 | 2.28E-18 |
| rs12138231 | 1 | A | T | 0.0669949 | 0.8503 | 0.0473 | 7.99E-09 |
| rs12151767 | 2 | A | G | -0.0611045 | 0.3988 | 0.0345 | 1.31E-12 |
| rs12285419 | 11 | A | C | 0.0849045 | 0.1528 | 0.0476 | 1.05E-14 |
| rs12293670 | 11 | G | A | -0.0704957 | 0.3303 | 0.0358 | 1.56E-14 |
| rs12303743 | 12 | C | G | 0.0874988 | 0.08932 | 0.0593 | 1.59E-09 |
| rs12489270 | 3 | C | T | 0.0579046 | 0.3586 | 0.0352 | 7.47E-11 |
| rs12652777 | 5 | C | T | -0.0487997 | 0.5322 | 0.0338 | 1.52E-08 |
| rs12712510 | 2 | C | T | -0.0574006 | 0.5148 | 0.0338 | 5.14E-11 |
| rs12771371 | 10 | A | G | -0.0524027 | 0.2566 | 0.0386 | 1.94E-08 |
| rs12833624 | 12 | T | C | 0.0501992 | 0.2999 | 0.037 | 2.77E-08 |
| rs12877581 | 13 | C | G | 0.0596014 | 0.3226 | 0.0363 | 1.80E-09 |
| rs12883788 | 14 | T | C | 0.0613011 | 0.4498 | 0.0342 | 1.86E-12 |
| rs13011472 | 2 | G | C | 0.070401 | 0.4487 | 0.0339 | 4.28E-16 |
| rs13016542 | 2 | C | T | -0.0883039 | 0.1469 | 0.0476 | 8.28E-12 |
| rs13107325 | 4 | T | C | 0.158703 | 0.01411 | 0.144 | 2.90E-21 |
| rs13233308 | 7 | T | C | -0.0487044 | 0.4849 | 0.0337 | 1.75E-08 |
| rs132582 | 22 | T | C | -0.0509972 | 0.5091 | 0.0337 | 3.26E-09 |
| rs1427633 | 4 | C | G | -0.0483043 | 0.5846 | 0.0343 | 4.10E-08 |
| rs1430894 | 3 | T | C | 0.0532953 | 0.5645 | 0.0339 | 6.15E-10 |
| rs145071536 | 1 | C | T | 0.0851005 | 0.1697 | 0.0452 | 1.62E-12 |
| rs1451488 | 2 | G | A | 0.0708947 | 0.6192 | 0.0347 | 4.47E-16 |
| rs149165 | 16 | G | T | -0.0481995 | 0.4239 | 0.0343 | 3.00E-08 |
| rs1540840 | 14 | C | G | -0.0556996 | 0.4877 | 0.0342 | 2.21E-09 |
| rs1593304 | 7 | G | A | 0.0641013 | 0.8751 | 0.0516 | 7.45E-09 |
| rs1604060 | 3 | G | A | 0.0772051 | 0.7929 | 0.0417 | 3.24E-08 |
| rs1611236 | 6 | A | G | -0.0551035 | 0.2234 | 0.0437 | 8.47E-09 |
| rs1615350 | 12 | T | C | -0.0736036 | 0.7874 | 0.0413 | 4.92E-14 |
| rs167924 | 3 | G | A | 0.0501992 | 0.5826 | 0.0342 | 2.34E-08 |
| rs16851048 | 1 | C | T | 0.0744973 | 0.1484 | 0.0475 | 4.15E-12 |
| rs16867571 | 5 | G | A | -0.0657035 | 0.2179 | 0.041 | 2.68E-10 |
| rs17194490 | 3 | T | G | 0.0781994 | 0.1402 | 0.0485 | 1.80E-11 |
| rs17731 | 10 | A | G | 0.0523992 | 0.2633 | 0.0382 | 4.37E-09 |
| rs1860002 | 12 | T | C | -0.0837987 | 0.5518 | 0.034 | 1.04E-21 |
| rs187557 | 5 | T | C | -0.0666956 | 0.8873 | 0.0533 | 2.03E-08 |
| rs1881046 | 2 | T | G | -0.0507026 | 0.2943 | 0.0371 | 3.39E-08 |
| rs1892346 | 1 | A | T | 0.0484027 | 0.5746 | 0.0341 | 3.56E-08 |
| rs1901512 | 5 | C | T | -0.058401 | 0.7276 | 0.0379 | 5.72E-10 |
| rs1914399 | 7 | G | C | -0.0491044 | 0.5009 | 0.0337 | 1.40E-08 |
| rs1915019 | 8 | G | A | -0.0570984 | 0.7844 | 0.041 | 6.57E-09 |
| rs1953205 | 14 | A | T | 0.0499048 | 0.4891 | 0.034 | 2.21E-08 |
| rs2053079 | 19 | G | A | 0.0598986 | 0.2598 | 0.0386 | 3.01E-09 |
| rs2078266 | 9 | G | A | -0.0696007 | 0.8325 | 0.0464 | 2.94E-08 |
| rs215412 | 4 | A | G | 0.0577033 | 0.3418 | 0.0355 | 2.69E-10 |
| rs2167378 | 2 | T | C | -0.0648978 | 0.382 | 0.0347 | 7.30E-14 |
| rs217336 | 6 | A | C | -0.0503033 | 0.4863 | 0.0337 | 8.05E-09 |
| rs2333321 | 4 | G | A | -0.0712038 | 0.8017 | 0.0423 | 1.25E-11 |
| rs2381411 | 9 | C | T | 0.050399 | 0.442 | 0.0341 | 1.25E-08 |
| rs2455415 | 13 | T | C | 0.0494949 | 0.3675 | 0.0349 | 1.69E-08 |
| rs2456020 | 15 | T | C | -0.0815984 | 0.2866 | 0.0371 | 1.13E-15 |
| rs2514218 | 11 | T | C | -0.0704957 | 0.2025 | 0.0422 | 1.35E-14 |
| rs2696466 | 17 | G | A | -0.0611986 | 0.3748 | 0.035 | 2.64E-11 |
| rs2710323 | 3 | C | T | -0.0784044 | 0.4849 | 0.0337 | 1.23E-19 |
| rs2815731 | 6 | A | C | -0.0600033 | 0.441 | 0.0339 | 4.39E-11 |
| rs2909457 | 2 | A | G | -0.0489997 | 0.6464 | 0.0353 | 1.48E-08 |
| rs2999392 | 14 | T | C | 0.0517987 | 0.7187 | 0.0375 | 3.05E-08 |
| rs308697 | 3 | A | C | -0.0501036 | 0.3652 | 0.035 | 8.83E-09 |
| rs34555420 | 6 | G | C | -0.168696 | 0.01532 | 0.1422 | 1.54E-22 |
| rs34555420 | 6 | T | G | -0.168696 | 0.04694 | 0.0873 | 1.54E-22 |
| rs35351411 | 15 | C | A | 0.0635044 | 0.4823 | 0.0338 | 2.21E-13 |
| rs35426637 | 7 | T | G | -0.0622985 | 0.3477 | 0.0354 | 2.15E-11 |
| rs35734242 | 4 | C | T | 0.050704 | 0.4544 | 0.0338 | 1.37E-08 |
| rs3739118 | 2 | A | G | -0.057004 | 0.2817 | 0.0374 | 2.36E-09 |
| rs3770754 | 2 | G | C | -0.052896 | 0.4208 | 0.0342 | 5.35E-09 |
| rs3791710 | 2 | C | T | -0.0600033 | 0.1902 | 0.0431 | 3.02E-08 |
| rs3795310 | 1 | T | C | -0.0509972 | 0.3554 | 0.0353 | 5.75E-09 |
| rs3802924 | 11 | C | A | -0.0736036 | 0.2888 | 0.0373 | 9.58E-12 |
| rs3814883 | 16 | T | C | -0.0670977 | 0.4119 | 0.0342 | 1.58E-14 |
| rs3824451 | 9 | C | T | 0.0655951 | 0.133 | 0.0495 | 2.54E-08 |
| rs4129585 | 8 | C | A | -0.0749962 | 0.5967 | 0.0343 | 5.11E-18 |
| rs4575535 | 16 | G | A | 0.0557982 | 0.6212 | 0.0349 | 5.77E-09 |
| rs4632195 | 18 | T | C | 0.0471964 | 0.4684 | 0.0338 | 4.59E-08 |
| rs4636654 | 11 | A | G | -0.0483043 | 0.4219 | 0.0342 | 4.89E-08 |
| rs4653164 | 1 | T | C | 0.0511038 | 0.6069 | 0.0344 | 3.08E-08 |
| rs4700418 | 5 | G | C | 0.0701972 | 0.474 | 0.0337 | 5.37E-16 |
| rs4702 | 15 | A | G | -0.0843044 | 0.5289 | 0.0339 | 2.79E-21 |
| rs4766428 | 12 | T | C | 0.0750038 | 0.4408 | 0.0339 | 3.93E-17 |
| rs4779050 | 15 | G | T | -0.0579953 | 0.5874 | 0.0342 | 7.27E-11 |
| rs4812325 | 20 | A | G | 0.0719042 | 0.616 | 0.0347 | 8.96E-16 |
| rs4921741 | 8 | G | A | 0.0559991 | 0.3274 | 0.036 | 1.21E-08 |
| rs498591 | 9 | T | A | 0.0724954 | 0.1467 | 0.0478 | 2.11E-09 |
| rs500102 | 9 | C | T | -0.0517002 | 0.5375 | 0.0338 | 4.87E-09 |
| rs505061 | 9 | A | C | 0.0534957 | 0.4656 | 0.0339 | 5.80E-10 |
| rs56205728 | 15 | A | G | 0.0630037 | 0.2399 | 0.0398 | 1.01E-10 |
| rs56335113 | 1 | G | A | -0.064701 | 0.7055 | 0.037 | 6.02E-12 |
| rs57433322 | 17 | G | C | -0.0830996 | 0.1556 | 0.0464 | 1.99E-09 |
| rs5751191 | 22 | C | T | 0.0655951 | 0.5764 | 0.034 | 3.00E-14 |
| rs58120505 | 7 | C | T | -0.089603 | 0.35 | 0.0353 | 2.24E-24 |
| rs6001259 | 22 | T | C | 0.1915 | 0.04445 | 0.0812 | 3.70E-08 |
| rs6010045 | 22 | C | T | 0.0548998 | 0.783 | 0.0409 | 7.44E-09 |
| rs61937595 | 12 | T | C | -0.130098 | 0.1411 | 0.0488 | 1.15E-15 |
| rs62018952 | 15 | C | T | 0.0584027 | 0.8417 | 0.0463 | 1.94E-09 |
| rs62183855 | 2 | C | A | -0.0660967 | 0.07228 | 0.0649 | 2.66E-09 |
| rs634940 | 6 | T | G | 0.0663962 | 0.2105 | 0.0414 | 1.78E-11 |
| rs6482437 | 10 | C | A | 0.0989036 | 0.8641 | 0.0493 | 3.33E-12 |
| rs6538539 | 12 | T | G | -0.0567961 | 0.488 | 0.0337 | 4.43E-11 |
| rs6546857 | 2 | G | A | 0.0603978 | 0.2203 | 0.0404 | 2.74E-09 |
| rs6549963 | 3 | C | T | -0.0483043 | 0.4036 | 0.0345 | 4.31E-08 |
| rs6673880 | 1 | G | A | 0.062301 | 0.5764 | 0.0342 | 7.19E-12 |
| rs6715366 | 2 | A | G | 0.0540972 | 0.2718 | 0.0379 | 2.49E-08 |
| rs6721531 | 2 | T | A | -0.0517002 | 0.179 | 0.0443 | 1.47E-08 |
| rs6798742 | 3 | G | A | 0.0610991 | 0.3084 | 0.0364 | 4.57E-11 |
| rs6943762 | 7 | C | T | -0.105098 | 0.1268 | 0.0507 | 1.57E-15 |
| rs6974218 | 7 | C | A | -0.0548953 | 0.4871 | 0.0338 | 6.80E-10 |
| rs6984242 | 8 | A | G | -0.0546965 | 0.5692 | 0.034 | 3.85E-10 |
| rs708228 | 11 | T | C | 0.0527997 | 0.3656 | 0.0349 | 6.56E-09 |
| rs7112616 | 11 | C | T | -0.0522034 | 0.4659 | 0.0339 | 1.52E-09 |
| rs7113199 | 11 | C | A | -0.0522983 | 0.6853 | 0.0363 | 2.80E-08 |
| rs7251 | 19 | G | C | -0.0641009 | 0.268 | 0.0381 | 8.29E-12 |
| rs72802868 | 5 | T | G | -0.0691995 | 0.294 | 0.037 | 4.55E-13 |
| rs728055 | 7 | A | T | -0.0673969 | 0.3474 | 0.0354 | 8.85E-14 |
| rs72943392 | 11 | C | G | 0.0534957 | 0.3147 | 0.0362 | 2.39E-08 |
| rs72986630 | 19 | T | C | 0.112296 | 0.06253 | 0.0699 | 3.59E-10 |
| rs73229090 | 8 | A | C | -0.102602 | 0.0954 | 0.0574 | 4.34E-13 |
| rs73292401 | 17 | A | T | 0.0676045 | 0.1588 | 0.0461 | 5.48E-10 |
| rs7515363 | 1 | T | C | -0.0535029 | 0.5705 | 0.0341 | 1.84E-09 |
| rs7575796 | 2 | G | A | -0.0963006 | 0.2264 | 0.0409 | 2.07E-08 |
| rs7634476 | 3 | G | A | 0.0577033 | 0.4605 | 0.0339 | 5.46E-11 |
| rs7647398 | 3 | T | C | -0.0774979 | 0.1363 | 0.049 | 1.07E-12 |
| rs778371 | 2 | G | A | 0.0806029 | 0.2707 | 0.0379 | 1.49E-17 |
| rs7798283 | 7 | G | T | -0.074003 | 0.1051 | 0.0551 | 3.49E-08 |
| rs79210963 | 7 | C | T | 0.0856015 | 0.1065 | 0.0549 | 4.14E-10 |
| rs79445414 | 8 | C | T | 0.1234 | 0.07047 | 0.0661 | 2.80E-08 |
| rs8055219 | 16 | A | G | 0.0665031 | 0.2351 | 0.0397 | 5.69E-11 |
| rs9304548 | 18 | A | C | -0.0567016 | 0.7279 | 0.0381 | 1.59E-08 |
| rs9318627 | 13 | C | A | -0.0611986 | 0.3446 | 0.0354 | 4.35E-12 |
| rs9454727 | 6 | G | A | -0.054403 | 0.2837 | 0.0374 | 3.35E-08 |
| rs9461916 | 6 | C | T | 0.0532953 | 0.5702 | 0.0342 | 1.64E-09 |
| rs9636107 | 18 | G | A | 0.0698969 | 0.4599 | 0.0338 | 5.11E-16 |
| rs9687282 | 5 | G | T | 0.0525994 | 0.299 | 0.0368 | 7.33E-09 |
| rs9876421 | 3 | T | C | 0.0625033 | 0.3133 | 0.0365 | 9.19E-12 |
| rs2332700 | 14 | G | C | -0.0750982 | 0.7301 | 0.0381 | 3.88E-14 |
| rs13195636 | 6 | C | A | -0.210504 | 0.05004 | 0.0876 | 6.55E-40 |

**Table S2 Instrumental variables used in MR analysis of the association between**

**LP and schizophrenia**

| SNP | chr | EA | OA | BE | eaf | se | pval |
| --- | --- | --- | --- | --- | --- | --- | --- |
| rs138365496 | 12 | A | C | 0.4806 | 0.036 | 0.0245 | 2.21E-06 |
| rs139504901 | 10 | G | C | 0.7302 | 0.017 | 0.0373 | 3.27E-06 |
| rs17170849 | 7 | C | T | 0.7256 | 0.02 | 0.0322 | 2.17E-06 |
| rs193769 | 16 | C | T | -0.1707 | 0.665 | 0.0092 | 4.66E-06 |
| rs2399594 | 16 | G | A | 0.16 | 0.385 | 0.0089 | 2.64E-06 |
| rs72720607 | 4 | A | G | 0.7655 | 0.026 | 0.0299 | 1.15E-06 |
| rs72764621 | 5 | T | C | 0.3671 | 0.02 | 0.0393 | 4.18E-06 |
| rs9671648 | 14 | C | T | 0.3535 | 0.061 | 0.0182 | 7.65E-07 |

Supplementary Table 1: SNP: single nucleotide polymorphism; EA: effect allele; OA: non-effect allele; Chr: chromosome; EAF: effect allele frequency; Beta was obtained by allele-related effects; SE: standard error. Beta, SE, and Pval are SNP summary statistics; LP: lichen planus.
